# “*They don't have the luxury of time*”: interviews exploring the determinants of public health research activity that contextualise embedded researcher roles in local government

**DOI:** 10.1186/s12961-024-01162-2

**Published:** 2024-07-31

**Authors:** Rachael C. Edwards, Dylan Kneale, Claire Stansfield, Sarah Lester

**Affiliations:** Evidence for Policy and Practice Information Centre, UCL Social Research Institute, Institute of Education, University College London, Gower Street, London, WC1E 6BT United Kingdom

**Keywords:** Embedded researcher, Evidence use, Local government, Public health, Research activity

## Abstract

**Background:**

Embedded researchers are a novel intervention to improve the translation of research evidence into policy and practice settings, including public health. These roles are being implemented with increasing popularity, but they often lack clear evaluative frameworks. Understanding initial levels of research activity, including associated barriers and opportunities, is essential to developing theories of change and thus shaping the roles and defining expectations. We aimed to identify the principal determinants of research activity in public health that contextualise embedded researcher roles, including attributes of the embedded researcher themselves.

**Methods:**

We undertook seventeen semi-structured interviews with embedded researchers in diverse public health settings in English local government. Interviews were analysed using thematic analysis.

**Results:**

We identified thirteen interlinked determinants of research activity within local government public health settings. Research and interpersonal skills, as well as pre-existing connections and knowledge within local government, were highly valued individual attributes for embedded researchers. Resource deficiencies (funding, time, and infrastructure) were primary barriers to research activity, whereas a strong local appetite for evidence informed decision making presented a valuable opportunity. However, there was inconsistencies across public health teams relating to perceptions of what constituted “research” and the resources that would be required.

**Conclusions:**

Our results suggest that successful embedded researchers will have equally strong research and communication skills and should be offered mentorship and clear career progression pathways. Perceptions of research within local government are closely linked to resource deficiencies and senior endorsement. Embedded researchers could benefit from taking the time to develop locally contextualised knowledge of this research culture. Theories of change for embedded researchers should conceptualise the interconnections across individual, interpersonal, and organisational barriers and opportunities underlying local government research activity. Further research is needed to identify methods for exploring the influence of embedded researchers as well as to unpack the stages of research activity within local government and the associated behaviours.

## Background

In recent decades, researchers have been increasingly interested in the mechanisms underlying the translation of research into practice [1, 2]. This concern was born largely from the recognition of an enduring research-implementation gap whereby academic research is not translated into decision-making within policy and practice settings [3]. In public health, bridging this gap is critical to ensuring that depleted public funds are efficiently allocated to address rising health inequalities. However, the processes through which research diffuses into public health decisions are highly complex, non-linear, and constrained by a variety of barriers [4].

The literature has identified a wide range of factors that inhibit research activity within public health settings [5–7]. For example, through a systematic scoping review of the literature on public health decision making processes, Kneale, Rojas-García [4] identified several barriers to research evidence use including a lack of access to and applicability of academic research. Similarly, the National Institute for Health and Care Research (NIHR) recently funded studies across several local authorities in England to identify barriers and enablers of research activity [8]. These studies identified a variety of constraints both internal and external to local authorities including capacity limitations, misalignment of research timelines between local government and academia, and a lack of consensus on what constitutes research. To address such obstacles and improve cultures of evidence use in policy and practice, embedded researchers are increasingly being adopted within public health and a variety of other settings as a novel intervention at the research-policy interface [9–11].

Drawing from existing definitions [e.g. 12, 13], we have derived a set of principles for defining an embedded researcher [14]. These principles identify attributes shared by embedded researchers across diverse settings, while also embracing the variety of forms the roles can take. Broadly, embedded researchers are roles which are co-affiliated with a research and policy or practice setting to enable research activity and use. As such, they act as change agents and are more than an occasional collaborative relationship. Rather, they have continual engagement with a host organisation who has joint influence over their aims and activities. In the context of embedded researchers, *research activity* should be conceptualised in the broadest sense to reflect the wide variety of activities they undertake (e.g., co-production, capacity building, supporting research use) [15].

Embedded researchers are well placed to address barriers to research activity in public health as they can, among other things, improve the local relevance of research and enhance local buy-in through becoming immersed and building trust within a host organisation [11, 16]. In recognition of this value, researchers are increasingly being embedded as change agents within public health practice [14]. A growing number of examples have since emerged demonstrating how embedded researchers can activate incremental change in research cultures through, for example, “growing networks, becoming a local expert and champion, and enhancing evidence fluency (the skills needed to source and interpret evidence) or curiosity about evidence and research” [17 pg. 3, 18, 19].

Despite their identified potential in public health, embedded researchers are still relatively novel in these settings, often quite exploratory and lacking clear objectives and monitoring frameworks [10, 20]. Understanding initial levels of research activity, including associated barriers and opportunities, is essential to developing theories of change for embedded researcher interventions and thus shaping the roles and defining expectations. However, given the numerous determinants of research activity in public health, investigating the local research context to inform embedded researcher interventions has the potential to be a highly onerous process. As such, the capacity constraints common in public health settings [6] present a significant barrier to the efficient design of embedded researcher roles, including aims and expectations. Identifying the determinants of research activity which are likely to be most relevant to embedded researchers could streamline this initial investigation.

A growing body of work has explored barriers and opportunities underlying embedded researcher roles [9, 11, 21]. For example, Coates and Mickan [1], surveyed over 100 ‘embedded researchers’ in Australian healthcare organisations to identify challenges and opportunities. They found, for example, that research was not sufficiently valued within healthcare organisations, but that access to research colleagues and mentors was a primary enabler. Some of this work has focused on procedural barriers and opportunities for embedded researchers such as those relating to attributes of the roles and strategies for becoming embedded within a host team [9, 22, 23]. This body of research has significantly advanced our understanding of embedded researcher interventions and many of the inhibiting and enabling factors align with the determinants of research activity within public health more generally. However, much of this work has emerged from clinical healthcare settings, and to our knowledge, there has been no comprehensive summary of the determinants of research activity in public health settings in the context of embedded researchers.

Through interviews with a diverse cohort of embedded researchers in English local government, we aim to identify the principal determinants of research activity in public health that contextualise embedded researcher roles. Among other benefits, investigating these attributes and the extent to which they present a barrier or opportunity to research activity at the inception of embedded researcher posts will assist with defining expectations, priorities, and theories of change for these interventions. We explore these determinants across three distinct, but connected layers of influence:

i. Individual: Attributes of the embedded researcher
ii. Interpersonal: Attributes of the embedded researcher’s local government colleagues
iii. Organisational: Attributes of the local government system

Organisational and interpersonal determinants of research activity have typically been grouped under “contextual factors” within the literature and, as such, distinct analysis of these layers of influence provides a unique perspective. We include individual attributes relating to the embedded researcher themselves such as their own knowledge, skills, and experience, as these factors intersect with attributes of the local government and should similarly be considered when defining objectives embedded researchers can be expected to achieve and identifying the types of support they will require in this process.

## Methods

### Case study: clinical research network research practitioner posts

For this research, we interviewed embedded researchers who were a part of a programme of work funded by the NIHR through its Clinical Research Network (CRN). Referred to as Public Health Local Authority Research Practitioners (PHLARPs), these embedded researchers were based across twenty-three English local authority (LA) public health settings who had bid into the CRN to receive funding for the posts (some PHLARPs were embedded in multiple LAs, while in other cases LAs supported multiple PHLARPs as part of job share arrangements). In the UK, LA public health teams set the direction of local policy and direct the delivery of local public health activity in cooperation with other LA departments and allied bodies such as Health and Wellbeing Boards [4].

The PHLARP roles were a part of a novel and exploratory set of interventions aimed at facilitating and enhancing public health cultures of research engagement and activity within local government. We undertook research to investigate various aspects of this programme. The present paper reports on one component of this broader programme of research [17].

The purpose of the PHLARP roles was to enable LA public health teams to build their research activity. The PHLARPs worked towards this overarching aim primarily through capacity building activity such as linking LA colleagues to research opportunities, building networks, supporting research projects, and facilitating training, although they also in some cases co-produced research with the LA [23]. In March 2020, the first two PHLARPs started in post and most of the remaining cohort followed in spring 2021. Originally, these positions were advertised as one-year contracts with a salary ranging between approximately £28,000–43,000. However, partway through this initial year, most posts were extended.

We conceptualise PHLARPs as embedded researchers as the structure and aims of the posts align with our set of principles defining embedded researcher roles [14]. In short, the roles were affiliated within a host LA team, while still maintaining links with an academic institution, such as a university or the CRN, and involved the broad aim of facilitating research activity. Despite being linked through overarching aims and objectives, the PHLARP roles were operationalised flexibly based on the local context including the local needs and priorities. This flexibility was an important aspect of the programme given the diversity of LAs in which PHLARPs were based. In practice, LA staff contributed to, and often led on the formulation of job descriptions, usually alongside an academic partner. The CRN-PHLARP programme thus presents a unique opportunity to explore contextual factors across embedded researchers with similar overarching aims but embedded within diverse LA settings.

### Recruitment and semi-structured interview protocol

The CRN provided our research team with contact details for most PHLARPs that were funded through their programme. We pilot tested our interview schedule with one of these PHLARPs in November 2021. This initial interview was included within our final sample as the schedule did not change significantly. We then contacted the remaining PHLARPs about their potential involvement and carried out interviews in spring 2022. All interviews were conducted online through either Zoom or Teams. Prior to starting each interview, we (i) informed participants of the anonymity of their responses, (ii) attained informed consent for their participation, and (iii) requested their consent to the use of an audio recorder. This research was approved by a University College London research ethics committee (REC1540).

The first section of the interview consisted of gathering basic details about the PHLARP’s roles including start date, contract length, weekly time allocation, and any shared responsibilities (e.g., if the post was a job share). We also collected details on their LA and academic affiliations. We then asked a range of open ended, semi-structured questions revolving around (i) the research culture within the LA and how this had changed over the course of the PHLARP’s time in post, (ii) factors the PHLARPs perceived to have enabled or hindered their activities and influence on research culture, and (iii) skills the PHLARPs had relied upon or needed to develop during the post. Throughout the interviews, we asked PHLARPs to provide illustrative examples to provide further context to their responses.

### Overview of participants

We conducted a total of seventeen interviews with PHLARPs which represents approximately seven-in-ten of all those originally recruited to the posts. All interviews were audio recorded, lasting an average of 49 min (range: 34–69 min), and manually transcribed.

PHLARP posts were highly diverse in relation to the structure of their roles and affiliations with academia and local government. At the time of our interviews, PHLARPs had been in their roles between six months and one and a half years. Of those interviewed, approximately half were full time for at least some of the duration of their post (*n* = 9). The remaining PHLARPs were part time, five of whom split their role as part of a job share. Just over half (*n* = 10) were primarily affiliated within a single layer of local government (e.g., London borough, city council) and the remainder had a remit to work across several local administrative units (e.g., a county council or different LAs). While most PHLARPs were affiliated primarily with a public health team or other department at a similar level within the LA, a few held strong connections with more senior bodies involved with overarching strategic decision making.

All PHLARPs held some level of dual affiliation across a LA and either a university or local CRN (one of our defining principles for embedded researchers), but the relative level of affiliation across these organisations varied substantially: a quarter (*n* = 4) were more closely affiliated with a university than the local government, half (*n* = 8) held weak affiliations with an academic organisation beyond the CRN, and just over a quarter (*n* = 5) held an equal level of dual affiliation across academia and local government. PHLARPs with greater levels of research experience generally held the strongest levels of affiliation with universities whereas those who were early in their career with respect to research tended to exhibit stronger levels of embeddedness within local government.

### Analysis

We analysed our transcripts through an inductive thematic analysis approach using NVIVO qualitative analysis software (released in March 2020) [24]. We applied the guidelines of Braun and Clarke [25] for thematic analysis. While reviewing the transcripts for accuracy, we compiled an initial list of codes relating to the determinants of research activity within the LA. Through two additional rounds of data review, we added to and modified this initial list and merged codes into final themes and sub themes. Finally, these codes were grouped into hierarchical, interrelated levels of influence within the LA: Individual—Attributes of the embedded researcher, Interpersonal—Attributes of the embedded researcher’s LA colleagues, and Organisational—Attributes of the local government system. For each theme (i.e., determinant of research activity), we reviewed the associated text to explore the context in which it was discussed as well as if it was framed as an opportunity, barrier, or both across PHLARPs. The primary analysis was performed by the lead author, with the second author double coding a randomly selected twenty five percent sample of the transcripts. Any disagreements in the coding were discussed. Once coding was finalised, we calculated thematic frequencies. Most PHLARPs in our sample were each connected with a distinct LA, but two individuals held their posts as a job share within a single LA. Nevertheless, we considered these PHLARPs to be distinct units of analysis for the purposes of calculating frequencies as they brought unique sets of experience to their roles and reflected individual perspectives.

## Results

**Fig. 1:**
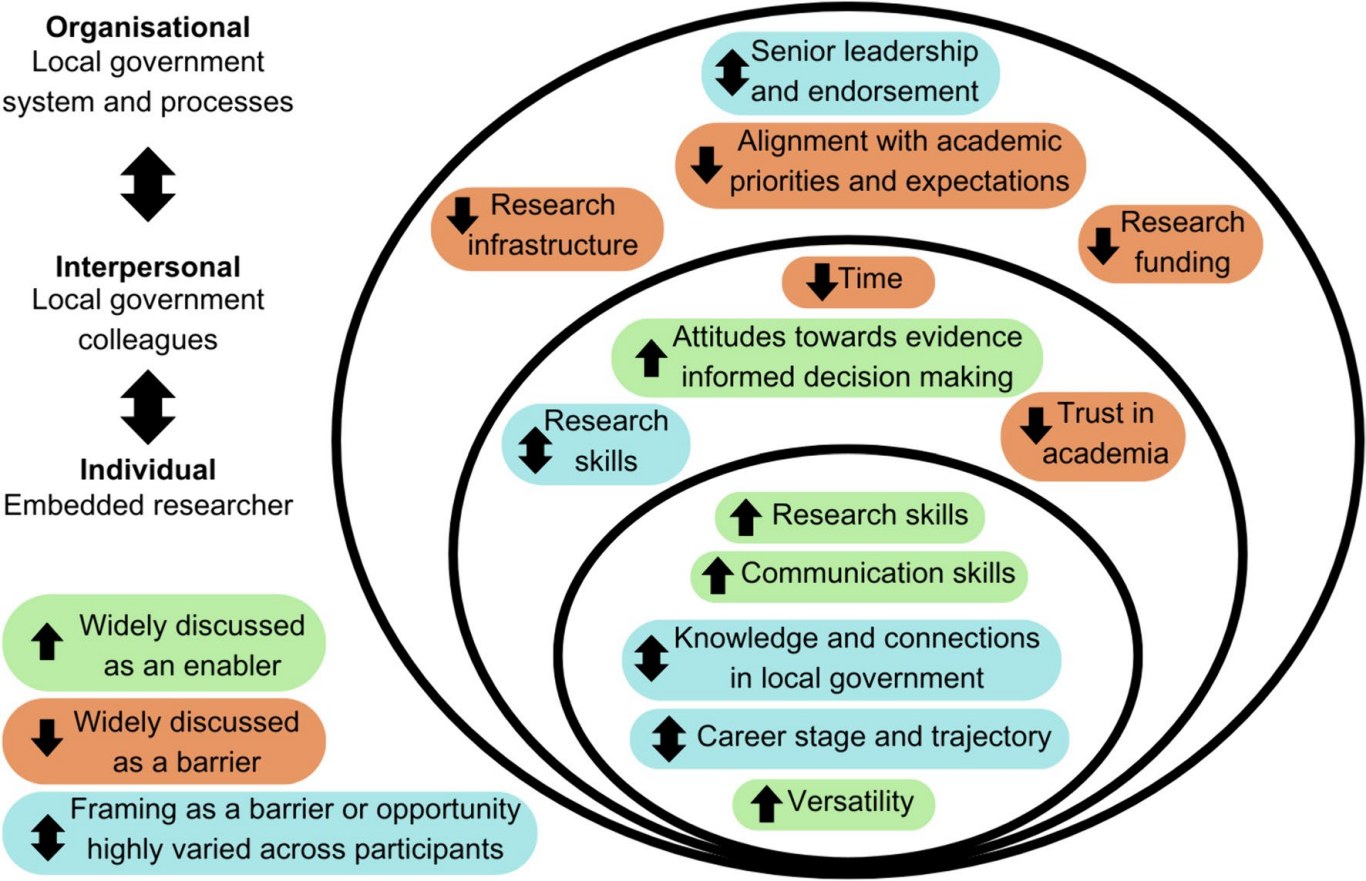
Nested, interconnected determinants of research activity in public health settings that contextualise embedded researcher roles

PHLARPs identified thirteen primary, interlinked factors that they perceived to underly levels of research activity within LA public health settings (Fig. 1). These factors are likely to be highly relevant to embedded researcher roles and could thus inform embedded researcher activity and theories of change. For some factors, their framing as either a barrier or opportunity varied substantially across LAs. For most, however, responses were relatively consistent with regards to whether it presented a hindrance or enabler of research activity. Although we have placed these determinants of research activity within multiple layers of influence on embedded researcher roles, our results also highlight many interconnections across factors and layers. These relationships suggest that effectively addressing barriers to LA research activity requires a holistic approach which embraces the inseparability of individual, interrelation, and organisational determinants.

### Individual: attributes of the embedded researcher

At the level of an individual embedded researcher, PHLARPs described five primary attributes that affected their ability to influence LA research activity. Most of these factors related to knowledge and skillsets that were valued in the role, but PHLARPs also described aspects of their career stage and trajectory that should be considered in the design of embedded researcher posts.

First, possessing research skills and experience was regularly described as a primary enabling factor for PHLARPs (*n* = 15). Indeed, most participants held research degrees and possessed a strong level of research experience. PHLARPs emphasised the value of research skillsets to their roles, including experience in writing research bids, formulating research questions, and data collection and analysis methods. For example, a participant stated that, “*I’ve used my skills and knowledge around behaviour change, my background from my PhD, to try and make sure [our activity is] theory based*”. Skills in co-production and patient and public involvement were described as particularly valuable by several participants, with many working to further develop these skills while in post. Those with less research experience spoke about the steep learning curve they underwent in their roles, and many described the value of the mentorship and support they received through academic supervision and/or working with colleagues with complementary skillsets.

Also emphasised by most PHLARPs was the value of communication and interpersonal skills (*n* = 15). Participants described how much of their role revolved around networking, collaboration, public engagement, and promoting research opportunities. As part of these activities, participants described the need to influence colleagues to, for example, take up such opportunities and (particularly at the leadership level) support research activity. Understanding the local context, including capacity limitations and perceptions about research, and applying this knowledge was emphasised as key to effective communication. For example, a PHLARP described how,

“*I think communication has probably been the most important [skill]. […] A lot of the gains that have been made in terms of research culture in the team have been about understanding people's perceptions about research and challenging them and facilitating better conversations about research and making people feel comfortable to express ignorance or lack of understanding so that we can help. And making people feel comfortable*”.

Many PHLARPs described how much of their time was spent translating information between diverse stakeholders, such as academic institutions and the LA, who often differ significantly in their language and priorities. Research and communication skills were often jointly applied, such as when engaging in co-production or public engagement.

Several PHLARPs spoke about the value of pre-existing knowledge of, and connections within, the LA (*n* = 11) which varied substantially. While some were already embedded within the LA team prior to starting in the post, others had no prior experience working with LAs such as one PHLARP who said, *“At first, I didn't understand a single word of what anyone spoke. [LAs are] like an alien place completely*”. Knowledge of the systems and language enabled PHLARPs to navigate the LA and its many layers and complex decision-making processes. Furthermore, understanding the various roles within the LA enabled PHLARPs to efficiently direct and make research enquires. Those with strong pre-existing connections discussed how these links sped up the processes of establishing their role and building trust, thereby accelerating their influence. For example, a PHLARP described the value of their connections in saying, “*I’m well networked into colleagues right across the Council. I know how to engage people, and what to do if I’m unable to engage people, how to influence on that to move things forward. That's been really useful*”.

Versatility was also emphasised by over half of the PHLARPs as an asset in their role (*n* = 10). These PHLARPs described the significant day-to-day variation within their role and often identified an enjoyment of this diversity. “*No two days are the same*” and “*I get bored easily*” were common sentiments. A PHLARP described this in saying how, “*it was just wonderful to be able to have something that would always keep me on my toes. Really diverse, incredibly enjoyable. Constantly learning different things*”. Related skills were also identified under this theme including autonomy and adaptability.

Nine PHLARPs voiced how the career stage and trajectory of embedded researchers are important elements to consider in the design of their posts. In particular, early career researchers require enhanced support, mentorship, and career development opportunities (e.g., publication opportunities). Many PHLARPs who entered their posts at a later career stage held permanent academic posts to return to when their LA roles came to an end. This was not the case for most early career PHLARPs. Partly because their livelihoods relied upon contract extensions, some of these early career PHLARPs expressed a heightened sense of urgency to prove themselves in the role and demonstrate influence. These early career PHLARPs viewed the exploratory nature of the roles and the lack of clear objectives with greater apprehension than their more established colleagues. The short-term nature of PHLARP contracts also presented a barrier to the establishment of trust with LA colleagues.

### Interpersonal: attributes of embedded researcher’s local government colleagues

PHLARPs described four primary determinants of research activity at an interpersonal level relating to their LA team and colleagues (e.g., skills, resources, knowledge, experience, perceptions). Most prominent among these themes was the capacity (time) constraints of LA staff which limited their engagement with both PHLARPs and research activity more broadly (*n* = 16). These time constraints were exacerbated by the Covid-19 pandemic and its ongoing effects across the health system and beyond. For example, one PHLARP lamented that, “*it has been a tough gig doing [the role] during the pandemic because of the fact there's so many competing pressures on local authority public health teams*”. Because of these pressures, it took longer for the PHLARPs to forge relationships within the LA.

LA colleagues often displayed an initial lack of interest in research activity which was perceived not as a lack of enthusiasm for evidence use, but rather as a symptom of feeling overwhelmed with other responsibilities (e.g., service delivery). Several PHLARPs described how these capacity constraints were reflected in the job descriptions of LA colleagues: “*Their job descriptions do not have space for research, and since it is not in their job role, any research that they are trying to undertake, it's an add-on. And that seems to be the biggest barrier, that they don't have the resources and they don't have the time to do it*”. PHLARPs spoke about how these time constraints inhibited their colleagues from taking up research opportunities such as applying for research funding as there was often no one available to lead on a bid: “*The amount of time that it takes to set up a research project is what we need, at least six months […] But they don't have the luxury of time*”. This theme is strongly linked to a deficiency of LA research funding (an organisational barrier—see below).

An equivalent number of participants described how positive perceptions of evidence informed decision making can enhance uptake of research opportunities among colleagues (*n* = 16). Encouragingly, participants widely identified a strong appetite for evidence informed decision making within the LA and a widespread understanding of the value of research evidence to public health decisions. For example, a PHLARP indicated that their “*contacts in the public health team were very embracing of research and what it can do in a practical sense and then what value it can add to decisions being made by local authorities and councils*”. However, PHLARPs also described how some of their colleagues viewed research involvement as something that was necessarily time consuming and costly. As such, PHLARPs needed to communicate and frame research opportunities as adaptations to existing workloads rather than add-ons. Additionally, perceptions of what was meant by “research” were not always shared. For example, a PHLARP described how “*The feedback was that [“research”] was too specific and academic a word and it wasn't very applicable in the local authority setting*”. Variation in understandings of research again highlight the value of strong communication skills to PHLARP roles.

Third, many PHLARPs discussed how research activity was influenced by existing levels of research knowledge and skills within the LA (*n* = 13). These research skills were described as varying widely both across and within LAs. For example, some PHLARPs described a strong level of research experience among their colleagues such as one participant who said, “*capacity is there in the sense that they are capable people. Most of them in public health, they have had their masters, so they've done a research thesis. Many have PhDs. Many supervise masters*”. Others, however, perceived that these skills were lacking within the LA, or existed only in pockets or on certain teams.

Finally, almost a quarter of PHLARPs identified trust in academia as an influencer of research activity within the LA, all of whom perceived that such trust was lacking (*n* = 4). These PHLARPs recounted examples from LA colleagues of negative prior experiences collaborating with academics such as cases where research did not benefit nor was shared with the LA. For example, a PHLARP described how, “*the mutual value isn't always clear. So, it might be a really good piece of research, but in practice, what does it actually mean for the Council in terms of the resources they have to put in and the benefit for them? I think sometimes true collaboration can be something that's a bit missing*”.

### Organisational: attributes of the local government system

Across the wider organisational context, PHLARPs described four primary factors that they perceived to influence research activity. Funding (e.g., infrastructure, staffing) was particularly critical (*n* = 13). Unfortunately, almost all those who spoke about this factor identified a significant research funding deficit. For example, a PHLARP described how, “*I think the City Council is low capacity because it's been underfunded, and it’s not been able to benefit from some previous calls for work in this area. I’d say it's behind*”. This lack of funding filters down to exacerbate capacity constraints identified at the interpersonal level and reflects the current trend of austerity, evidenced by a PHLARP who said, “*the general financial climate within local authorities has meant that we’ve had a number of significant restrictions over the last year or two within the Council which are responding to local government finances*”. A few PHLARPs raised concerns about growing research funding inequities across LAs and the difficulties they experienced when competing for research funding with capacity rich LAs who had more established cultures of research.

Second, many PHLARPs identified how research activity was influenced by the extent of alignment between the priorities and expectations of academia and the LA (*n* = 14). Most PHLARPs perceived that a stronger level of alignment was needed. A PHLARP described this in saying, “*everyone has their own agenda. The County Council is pushing a report or audit, or something to do with public health. Whereas the university just want to get publications out there […] There’s that misalignment in a sense and that makes it really hard to draw everyone together and get everyone working on the same page*”. Beyond misalignment of research priorities, PHLARPs also described differences between the research expectations of LAs and academic institutions such as timelines and ethics procedures. Given the fast pace and responsive nature of LAs, one PHLARP described how, “*The robust methodical planning and the timing of things [in academia] doesn't always align*”.

Several PHLARPs described how strong levels of senior leadership and endorsement of research can benefit research activity through a variety of pathways (*n* = 11). “*You need that consistent leadership at the very top*” emphasised one PHLARP. PHLARPs identified how a lack of senior leadership can lead to concerns that time spent on research activity will not be valued. Perceptions and appetite for research among senior colleagues would also filter down through the LA, described by a PHLARP who said, “*the culture within the Council is really top down. So, if the managers think doing research is expensive and time consuming, ultimately the people the manager is managing will think the same*”.

Finally, PHLARPs emphasised the value of research infrastructure for enabling research activity (*n* = 10). This infrastructure was almost always described as lacking within LAs. Examples of such infrastructure included ethics forms and procedures, access to the literature, research enabling software (e.g., for referencing and analysis), and research strategies. Additionally, a few PHLARPs spoke about a deficiency of information on research activity (including relevant contacts) for those wanting to collaborate with the LA on research opportunities.

## Discussion

We identified thirteen primary determinants of research activity in LA public health settings across three interrelated layers of influence. Investigating which factors inhibit and facilitate research activity in a local setting presents a critical step in the design of embedded researcher posts as this information serves to contextualise the roles and informs the design of evidence-based theories of change. The factors identified in this research present a useful starting point for such investigation as they are likely to be highly relevant to embedded researcher posts in LA public health settings. As an additional aid for the development of these posts, we have summarised our findings into a list of recommended, interrelated considerations for the design of embedded researcher posts and their underlying theories of change (Table 1).

**Table 1:** Interrelated determinants of research activity to be considered in the design of embedded researcher roles and associated theories of change

A) Factors to consider in the recruitment of embedded researchers	
Research skills	Do they possess a research degree? How many years of research experience do they have? Are they experienced in co-production and patient/public involvement?
Communication skills	Do they possess strong written and verbal communication skills? Do they have experience building networks and partnerships among diverse stakeholders? Are they able to adapt their communication method and style to a range of audiences?
Local authority knowledge and connections	Do they have any pre-existing connections within the local authority? Have they previously worked with/in a local authority? How well do they understand local authority systems and processes?
Versatility	Are they able to multitask and efficiently switch between projects and balance multiple priorities and timelines?
Career trajectory and stage	To what extent does their job security depend on this post? Do they have an established research career? What opportunities, including mentorship, do they need to continue advancing in their career?
B) Factors to consider when assessing the research readiness of the local authority workforce	
Capacity (time)	Is research included within local authority job descriptions? What is the existing capacity to take on new roles and responsibilities?
Perception of research	Is the value of research to public health decisions widely recognised? Is the breadth of ways in which research can be conducted and applied recognised or is research perceived in a predominantly academic sense? Is there a shared understanding of what constitutes “research”?
Research skills	Does the workforce possess research degrees and/or experience? What research skills are underdeveloped within the local authority (e.g., project development, data collection, analysis, interpretation)?
Trust in academia	Has the workforce had prior experience working with academia and what is their perception of this experience?
C) Factors to consider when assessing the research readiness of local government organisational structures and processes	
Research funding	How much funding does the local authority allocate to research activity (e.g., staffing, infrastructure, collaboration, training)?
Alignment with academia	To what extent do local authority priorities, timelines for research production, and ethic procedures align with academia?
Senior leadership and endorsement	Were senior members of the local authority involved with obtaining funding and/or in the design of the embedded researcher post? What forms of evidence are valued among senior local authority staff? Do senior members of the local authority widely communicate research opportunities and their support for research?
Research infrastructure	Does the LA have access to published literature? Does the LA have established ethics processes and procedures? Does the LA have access to research enabling software (e.g., for referencing and analysis)? Does the LA have a research strategy?

Our results suggest that successful candidates for embedded researcher posts should have equally strong research and communication skills. These two skillsets have been highlighted by other work on embedded researchers which suggest that both are necessary for success in these roles [26, 27]. The value of interpersonal skills aligns with the critical importance of establishing trust and the capacity building and co-production activity embedded researchers will likely undertake including networking, influencing, and building linkages across institutions. Knowledge of local government and existing connections within this system were also identified as valuable to the roles. As such, LAs should plan for a longer initial scoping phase if the embedded researcher lacks prior experience working within a LA context. It takes time for embedded researchers to become immersed within a host organisation and for trust to be established, particularly within a complex organisation such as a LA, and a growing body of literature provides guidance to assist embedded researchers during this phase of their role [9, 22, 23].

The career stage and trajectory of an embedded researcher is another key factor for LAs to consider in the design and recruitment for the posts. The contract length, for example, will be of particular concern for early career researchers, many of whom will not have permanent posts or established track records in academia. Short contracts are also likely to affect LA staff investment in the embedded researcher’s work. A growing body of research suggests that a lack of career progression pathways and insufficient recognition for capacity building activities and achievements present significant challenges for embedded researchers [28, 29]. LAs and academic institutions must thus collaborate to ensure that career progression pathways, mentorship, and professional development opportunities are provided and that achievements beyond traditional academic outputs are recognised and valued. For example, this could include accessing funding opportunities such as the NIHR’s Pre-Doctoral and Doctoral Local Authority Fellowships which support staff in developing the necessary skillsets and create links with academia.

Many of the barriers to research activity identified at the interpersonal and organisational levels align with those previously described within the embedded researcher literature in clinical settings. For example, capacity constraints [22, 30], a lack of sufficient research infrastructure [1], and a misalignment of academic and local government priorities [3, 16] have all been identified in healthcare contexts. Some of these barriers have also been identified in the context of embedded researchers in public health settings [31] and within public health literature more broadly [6, 8]. Our research adds to this work, suggesting that these barriers are likely to be highly relevant to embedded researcher roles in public health. Furthermore, through separating interpersonal from organisational determinants, this paper teases apart layers of influence on embedded researcher interventions while also highlighting their interconnectedness. While it is useful to identify individual determinants, our results suggest that consideration for this interdependence is equally as important when designing theories of change for embedded researchers.

Our findings also provide detail on how local perceptions of research-based evidence can enable and hinder embedded researchers in fostering a research active LA. Embedded researchers widely perceived there to be a strong appetite for evidence informed decision making across LA staff, a result that is not often discussed within the literature [31]. However, there were inconsistencies in what was perceived to constitute “research”, with many feeling that engagement with research would necessarily be highly resource intensive. Given the severe capacity constraints faced by public health teams, such perceptions presented an initial barrier to embedded researcher activity. A mismatch between academic timelines and LA activity perpetuated such assumptions. Therefore, we suggest that to constructively engage with LAs, embedded researchers and academics more broadly must recognise and work within existing capacity constraints through adopting and communicating a flexible and pragmatic conceptualisation of research. At the same time, we also suggest that research should be integrated within LA job descriptions and advocate for the value that protected time for research could add to LA public health outcomes. Given these complexities surrounding perceptions of research within policy and practice settings, we would caution authors to avoid an oversimplified narrative depicting research evidence as being undervalued in these contexts.

### Developing theories of change for embedded researcher interventions

Developing a strong, evidence informed theory of change presents a critical step in operationalising embedded researcher roles as this theory links embedded researcher activities and projects to the research barriers and behaviours they aim to influence. Identifying baseline levels of research activity and associated barriers prior to an embedded researcher being in post could provide an initial level of structure and direction for the role, clarity that is likely to be particularly valued by early career researchers. However, there are also benefits to be gained from embedded researcher involvement in this process. For example, undertaking a needs assessment provides embedded researchers the opportunity to build trust with colleagues, a key stage in becoming embedded within a host organisation [23]. As such, we suggest that theories of change for embedded researchers would benefit from progressive development, initially co-created by the home and host organisations, and continually adapted as the embedded researcher becomes familiar with the local context.

This research focused on identifying determinants of research activity (barriers and opportunities). As described above, existing levels of research activity itself should also be investigated to inform the aims and theories of change for embedded researcher posts. To avoid ambiguity, “research activity” should be clearly defined in the context of the specific embedded researcher intervention. While certain dimensions of research activity have been investigated within local government (e.g., use of research evidence in decision making, co-production) [7, 32], little work has explored the behaviours that constitute research activity more broadly or developed associated theories or typologies (but see [33]). For example, research activity within a LA could include the type of evidence LA staff base decisions on, how often research (including evaluation) is conducted, the rigour of research that is undertaken, and how often the LA collaborates with research institutions. More work is needed in this area to aid LAs in identifying desired behaviours and stages they can expect as they develop their research maturity.

As part of our wider programme of work on embedded researchers, the many interlinked barriers and opportunities identified in this research have informed the development of a logic model conceptualising the stages of embedded researcher interventions and surrounding contextual factors (see Appendix 2 in [17]). In addition to the present research, several other projects have fed into this model including a systematic review, documentary analysis, a diary study, a survey, and further qualitative research involving the PHLARPs and other programme stakeholders. This model links embedded researchers to change in individual (e.g., attitudes) and organisational (e.g., funding, infrastructure) determinants of research activity, as well as to longer term change in this activity itself. Through displaying change across multiple determinants within a single stage, the model reflects their interconnectedness. For example, it indicates how strengthening the research infrastructure will likely need to take place alongside action to enhance research curiosity and enthusiasm. Drawing on this model could present a valuable starting point in the development of theories of change for individual embedded researcher posts.

## Conclusions

This paper identified many interrelated determinants of public health research activity at the interpersonal and organisational levels that are relevant to embedded researcher interventions. We also highlighted the skillsets necessary for embedded researchers to have an influence over these determinants. When interpreting our results, it is important to recognise that the PHLARPs investigated in this study reflect a specific way of implementing embedded researcher interventions, albeit one that allows for high levels of flexibility. For example, capacity building was the primary activity for most of our participants, with less emphasis placed on research production. This prioritisation differs from some definitions and schemes which frame embedded researcher roles predominantly around co-production (e.g., [13]). Furthermore, most of our embedded researchers did not work as part of senior strategic decision-making contexts within the LA. It would be useful to investigate potential variation in the determinants of research activity across different levels of local government.

Further research is needed to explore methods of assessing the influence of embedded researchers as well as to clarify the behaviours that constitute research activity within local government. Variation in PHLARP responses suggests that while some LAs have relatively strong research infrastructure and support, others were relatively weak. Theories of change for embedded researchers should be contextualised within this existing research culture. This variation also presents an important consideration for funders if they are to avoid perpetuating research inequities. Indeed, LAs with strong research cultures can capitalise on funding successes through building their research capacity and other resources they allocate to additional funding opportunities. Funding organisations could thus consider more targeted funding streams to support LAs at different stages of research maturity.

The present research has focused on the barriers and facilitators of research activity within LAs. It is also necessary to acknowledge that academic institutions have an equally important role to play in facilitating the translation of research into practice. Indeed, the underutilisation of research evidence is as much a reflection of the ‘supply’ side as the ‘demand’ side. We have highlighted a few of these barriers such as the length of academic timelines which do not match the pace and need for research within LAs. A lack of salience has also been identified as a prominent barrier to the use of academic research within local government [34]. As such, academics must actively work alongside LAs to address barriers to evidence use. Co-production presents key mechanisms to address these barriers, collaborations that can be enabled through embedded research activity.

## Data Availability

Due to the qualitative nature of this research, participants of this study did not agree for their data to be shared publicly, so supporting data is not available.

## Abbreviations

CRN: Clinical Research Network
LA: Local authority
NIHR: National Institute for Health and Care Research
PHLARP: Public Health Local Authority Research Practitioner
